# Tactics analysis and evaluation of women football team based on convolutional neural network

**DOI:** 10.1038/s41598-023-50056-w

**Published:** 2024-01-02

**Authors:** Lechuan Shen, Zhongquan Tan, Zekun Li, Qikun Li, Guoqin Jiang

**Affiliations:** 1 School of Physical Education, Jianghan University, Wuhan City, 430000 China; 2 Department of Computer Science, Nanjing University, Nanjing City, 210000 China

**Keywords:** Engineering, Materials science, Mathematics and computing, Nanoscience and technology, Optics and photonics, Physics

## Abstract

In order to realize the process of player feature extraction and classification from multi-frequency frame-changing football match images more quickly, and complete the tactical plan that is more conducive to the game, this paper puts forward a method for analyzing and judging the tactics of women’s football team based on Convolutional Neural Network (CNN). By extracting the players’ performance in recent training and competition from continuous video frame data, a multi-dimensional vector input data sample is formed, and CNN is used to analyze the players’ hidden ability before the game and the players’ mistakes in different positions on the field to cope with different football schedules. Before the formal test, 10 games of 2021–2022 UEFA Women’s Champions League were randomly selected and intercepted to train the CNN model. The model showed excellent accuracy in the classification of image features of various football moves and goal angles, and the overall classification accuracy of each category exceeded 95%. The accuracy of classifying a single match is above 88%, which highlights the reliability and stability of the model in identifying and classifying women’s football matches. On this basis, the test results show that: according to the analysis of players’ personal recessive ability before the game, after model image recognition and comparison, the difference between the four scores of players’ personal recessive ability with CNN mode and the manual score of professional coaches was smaller, and the numerical difference was within the minimum unit value, and the numerical calculation results were basically the same. According to the analysis of players’ mistakes in different positions on the field, CNN was used to monitor the real-time mistakes. It was found that the two players in the forward position made the highest mistakes, and they were replaced by substitute players at 73.44 min and 65.28 min after the team scored and kept the ball, respectively. After the substitute players played, the team’s forward position mistake rate decreased obviously. The above results show that CNN technology can help players get personal recessive ability evaluation closer to professional evaluation in a shorter time, and help the coaching team to analyze the real-time events better. The purpose of this paper is to help the women’s football team complete the pre-match tactical training, reduce the analysis time of players’ mistakes in the game, deal with different opponents in the game and improve the winning rate of the game.

## Introduction

With the progress of computer technology, more and more professional football teams begin to use more convenient quantitative analysis and prediction techniques to assist teams to complete tactical analysis and decision-making^1,2^. Today’s football is divided into national team and club forms according to the types of football matches. The national team forms, such as the World Cup, Asian Cup, European Cup and Olympic Games, require the participants to be their own nationalities. Club-style competitions, such as Premier League, Bundesliga and Serie A, as well as higher-level competitions in which the top players participated in last season, require higher personal abilities of players and have more frequent internal personnel circulation than national team competitions. At present, the data of most sports matches need to be recorded and counted manually on the spot or by watching videos. Under normal circumstances, the whole time of a football match does not include rest and overtime, which requires 90 min. These long-term data statistics and recording greatly reduces the efficiency of team tactical analysis. Using convolutional neural network (CNN) to analyze the players’ hidden ability before the competition and the players’ mistakes in different positions on the field can greatly reduce the workload of individual statisticians and provide extremely effective help for the whole competition and the technical statistics of each athlete^3–5^. As the first sport in the world, football video annotation and analysis are a time-consuming task. Once it is automated, it will bring benefits to coaches, players and spectators^6,7^. In modern football environment, non-contact and camera-based multi-player detection and tracking has become a reality, and automatic motion recognition can improve players’ performance through symbol analysis^8–10^.

In recent years, CNN has been widely used in image classification, language processing and other aspects. How to apply deep CNN optimization to football tactical analysis and research has become a hot topic for scholars all over the world. In domestic research, Liu et al. proposed an automatic detection method based on CNN for the detection of multi-scale training actions in sports videos, and realized the accuracy of the detection model with a very small amount of labeled data^11^. Yao et al.^12^ used CNN combined with the learning of depth information to extract 2D spatial features from still images, which expanded the application scope of CNN from images to the use of time information for video analysis, and improved the performance of motion recognition on large-scale benchmarks. Chen and Wang analyzed the sports work in the competition. The research of sports action analysis technology based on video has important application value, and the analysis results can be fed back to coaches and data analysts in real time to help complete tactical analysis and technical selection^13^. In foreign research, Rongved et al. proposed an algorithm to automatically detect events in football videos using 3D CNN. The algorithm uses sliding window method to scan a given video to detect events such as goals, yellow cards/red cards and player substitutions. The results show that the event can be detected with high recall, low delay and accurate time estimation, and it performs better when it can accept less accurate time estimation^14^. Podgorelec et al.^15^ developed a method for image classification, which used fine-tuning of transfer learning to train CNN model, and used hyperparametric optimization based on differential evolution, which significantly improved the classification performance on the dataset composed of football moving images. Hassan et al. applied CNN to predict the winning or losing and attribute sensitivity of the 2018 World Cup. They thought that sports scientists could use a new neural network model based on radial function to adjust training, tactics and confrontation analysis to improve their performance^16^.

Football is a sport that requires teamwork, but meanwhile, factors such as strength, speed and skill also play a key role. There are significant physiological differences between male and female football players, and female players may have certain limitations in physical strength. Therefore, in order to ensure the efficient training of female football players, it is necessary to pay more attention to the cultivation and training of skills. In addition, meeting the time difference between daily training and competition is very important to improve the real-time training. In this context, this paper proposes and explores a method based on CNN, hoping to provide accurate training feedback in the shortest time, help women’s football teams complete pre-match tactical training, deal with different opponents in the game, and improve the winning rate of the game.

## Application of CNN model in tactical analysis and evaluation of women’s football team

### CNN

**Figure 1:**
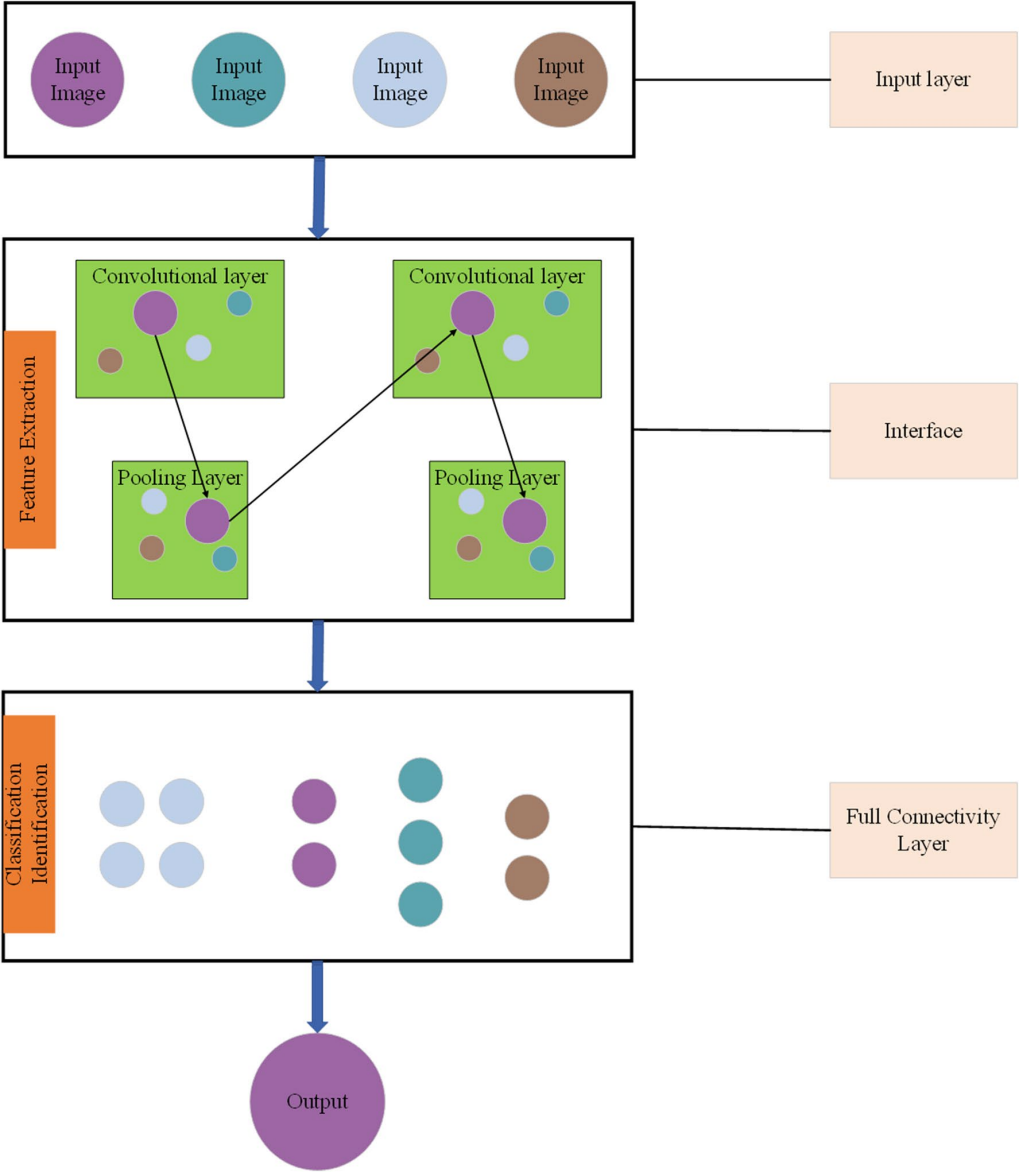
Basic structure diagram of CNN.

**Figure 2:**
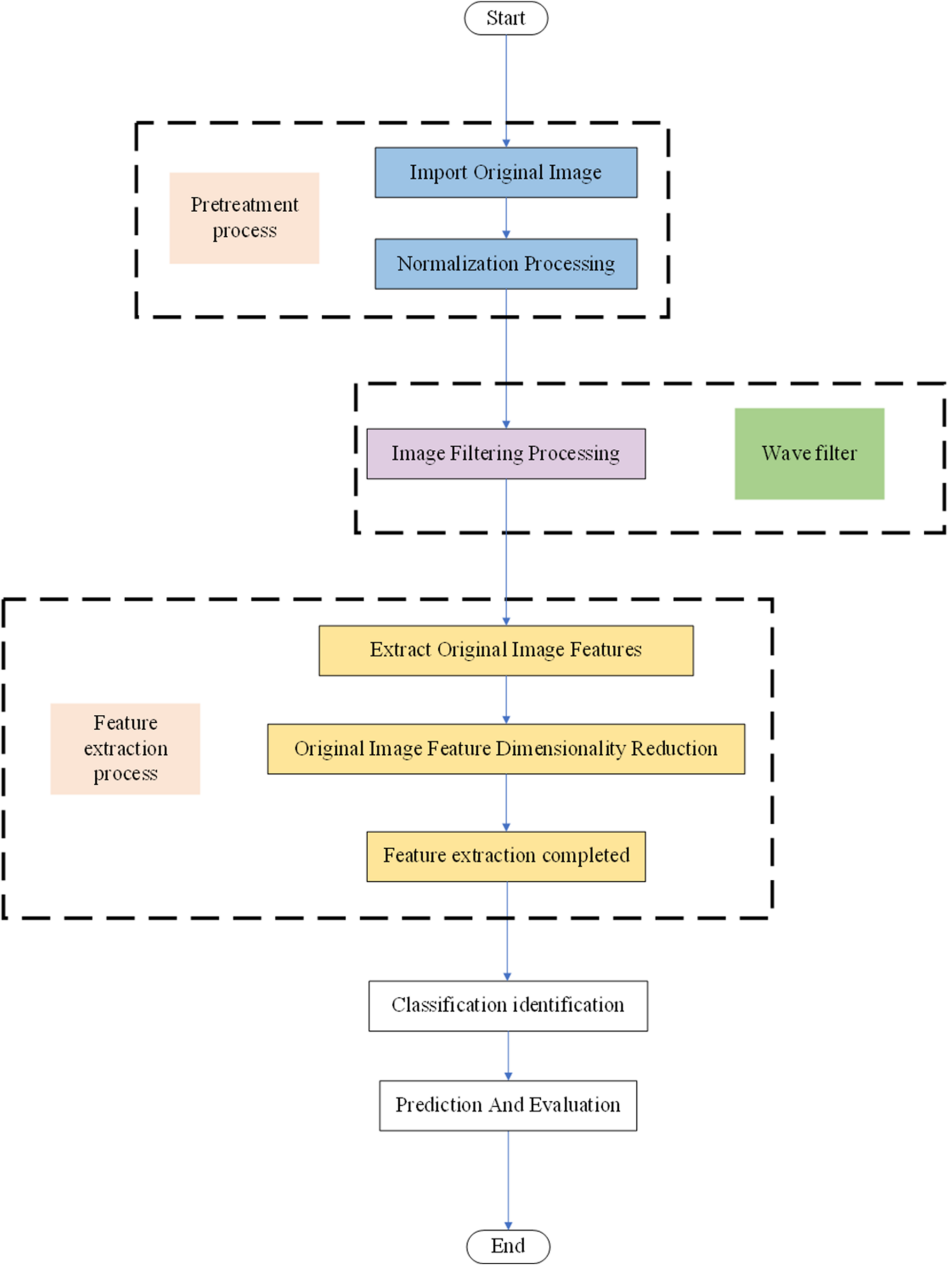
CNN flow chart.

CNN is a feedforward neural network, which includes convolution layer, pooling layer and fully connected layer^17^. Its artificial neurons can respond to the surrounding cells in a part of the coverage area, and the images input through the CNN model can be classified and output with a kind of information that most accurately conforms to the necessity of processing a specific form, which has excellent performance for large-scale image processing^18^. The layers in CNNs are composed of neurons and divided into three dimensions, which are called the dimensions of spatial input (height, width, and depth)^19^. By directly inputting the original data image, the complicated pre-processing of the image is reduced, and the feature extraction and classification process can be realized from the video image with multi-frequency frame changes more quickly^20^. The details are shown in Fig. 1:

CNN is one of the most important networks in the field of deep learning, and has made impressive achievements in many fields^21^. CNN, including but not limited to computer vision and natural language processing, can be used in multiple scenes^22,23^. CNN can be divided into four parts in the process of image feature extraction and classification, namely, input layer, middle layer, fully connected layer and output layer. The specific process is shown in Fig. 2:

### Function principle and realization method of each component of CNN

CNN is a kind of deep learning neural network specially used for image processing and pattern recognition. It has achieved remarkable success in the fields of image classification, object detection, image segmentation and so on^24^. In this paper, CNN is used to analyze the four hidden abilities of women football players, and their personal position mistakes are analyzed. The following is a description of each component of CNN network, their function principle and implementation method in the experiment:

1. Convolution layer
  Convolution layer is the core part of CNN, which is used to extract features from images. It performs convolution operation by sliding a convolution kernel on the input image, thus capturing local features at different positions. For each player’s picture, multiple convolution layers can be stacked, and each convolution layer contains multiple convolution kernels. These convolution kernels can learn different characteristics of each player, such as edge, texture, shape and so on. The output of convolution layer is called feature map, which contains the personal feature information of players in different positions in the image. The calculation equation of convolution operation can be expressed as:
  1 $$Z_{i,j} = \sum\nolimits_{m = 0}^{{KH^{ - 1} }} {\sum\nolimits_{n = 0}^{{KW^{ - 1} }} {\sum\nolimits_{k = 0}^{C - 1} {X_{{\left( {i S + m} \right),\left( {j S + n} \right),k}} *K_{m,n,k} } } }$$
  ${Z}_{i,j}$ represents the eigenvalue at position $(i,j)$ in convolution operation. $m,n$ represent the indexes on the height and width of convolution kernel respectively. $k$ represents the number of channels of the input feature map. ${X}_{\left(i S+m\right),\left(j S+n\right),k}$ represents the position $\left(i S+m\right),\left(j S+n\right)$ in the input feature map, and the channel is the value of $k$. ${K}_{m,n,k}$ represents the weight that the channel is $k$ at the position $(m,n)$ inside the convolution kernel.
  If the number of input channels is ${\text{I}}$, the number of output channels is ${\text{O}}$, the convolution kernel width is ${{\text{A}}}_{{\text{s}}}$, the convolution kernel height is ${{\text{A}}}_{{\text{l}}}$, and $+1$ means bias. ${\text{I}}\times {{\text{A}}}_{{\text{s}}}\times {{\text{A}}}_{{\text{l}}}$ represents the multiplication amount in a convolution operation, and ${\text{I}}\times {{\text{A}}}_{{\text{s}}}\times {{\text{A}}}_{{\text{l}}}-1$ represents the addition amount in a convolution operation. The parameter quantity of each convolution layer is ${\text{P}}$, and the operation quantity of each convolution layer is ${\text{F}}$, then:
  2 $${\text{P}} = {\text{O}} \times \left( {{\text{A}}_{{\text{s}}} {\text{*A}}_{{\text{l}}} {\text{*I}} + 1} \right)$$
  3 $${\text{F}} = \left[ {\left( {{\text{I}} \times {\text{A}}_{{\text{s}}} \times {\text{A}}_{{\text{l}}} } \right) + \left( {{\text{I}} \times {\text{A}}_{{\text{s}}} \times {\text{A}}_{{\text{l}}} - 1} \right) + 1} \right] \times {\text{O}} \times {\text{S}} \times {\text{L}}$$
  when ${\text{A}}_{{\text{s}}} = {\text{A}}_{{\text{l}}} = {\text{A}}$, the equation for calculating the parameter quantity of each convolution layer is:
  4 $${\text{P}} = {\text{O}} \times \left( {{\text{A}}^{2} {\text{*I}} + 1} \right)$$
  5 $${\text{F}} = 2 \times {\text{I}} \times {\text{A}}^{2} \times {\text{O}} \times {\text{S}} \times {\text{L}}$$
2. Activation function
  The activation function introduces nonlinear properties, which enables CNN to learn more complex feature mapping. In this paper, ReLU activation function is used. By applying activation function to the output of each convolution layer to introduce nonlinear transformation, the representation ability of the network can be enhanced to judge the individual level of players more accurately.
3. Pooling layer
  Pooling layer is used to reduce the spatial dimension of feature map, reduce the computational complexity, while retaining important features. Players’ personal ability analysis and error analysis in sports often contain high-dimensional feature map data. Adding a pooling layer after the convolution layer and reducing the spatial resolution of the feature map through pooling operation can help reduce the complexity of the model and improve the computational efficiency^25^. This helps to capture key information in players’ images to better evaluate their abilities and improve the efficiency of real-time monitoring of players’ status on the field. The calculation formula of pooling operation can be expressed as:
  6 $$Y_{i,j,k} = max_{l,t} *X_{{\left( {i S + l} \right),\left( {j S + t} \right),k}}$$
  ${Y}_{i,j,k}$ represents the eigenvalue at position $(i,j)$ in the pooling operation. $l,t$ represent the indexes on the height and width of the pooled window, respectively.
  The maximum pooling operation is studied with a 2 × 2 window and a step size of 2. The process of maximum pooling is to take the maximum value in the window as the output. The model grid description table is shown in Table 1:

**Table 1:** Model grid description table.

Layer type	Input size	Output size	remark
Input layer	4 × 4	4 × 4	Input matrix
Convolution layer	4 × 4	3 × 3	3 × 3 convolution kernel, step size 1, fill 0
Pooled horizon	3 × 3	2 × 2	2 × 2 window with step size 2
Convolution layer	2 × 2	1 × 1	3 × 3 convolution kernel, step size 1, fill 0
Output layer	1 × 1	1 × 1	Final output
4. Fully connected layer
  The fully connected layer transforms the feature map extracted from the convolution layer into the final classification result and ability score. Each neuron is connected with all neurons in the previous layer for integrating feature information and making classification decisions. Adding a fully connected layer at the top of the network, each neuron can represent a score of a specific ability, including the scores of observations and analysis ability, understanding and judgment ability, control and coordination ability and passing innovation ability, which can be used to evaluate the player’s personal ability level. The calculation equation of fully connected operation can be expressed as:
  7 $$Y_{i} = \sum\nolimits_{r = 0}^{D - 1} {X_{r} *W_{i,r} + M}$$
  ${Y}_{i}$ represents the output value at position $i$. $r$ represents the neuron index of the previous layer. ${X}_{r}$ represents the value of an element in the output feature vector of the previous layer at position $r$. ${W}_{i,r}$ represents the weight of connecting the $r$-th input feature to the $i$-th output neuron. $M$ represents the bias of the output neuron. If the weight number of neurons in the ${\text{M}}$-th layer is ${\text{B}}\times {\text{M}}$, the equation for calculating the parameters of the fully connected layer is:
  8 $${\text{P}} = \left( {{\text{B}} + 1} \right) \times {\text{M}} = {\text{B}} \times {\text{M}} + {\text{M}}$$
  ${\text{B}}$ represents the amount of multiplication, ${\text{B}}-1$ represents the amount of addition, $+ 1$ represents bias, and $\times {\text{M}}$ represents the calculation of the value of ${\text{M}}$ neurons, so the calculation formula of the amount of operation of the fully connected layer is:
  9 $${\text{F}} = \left[ {{\text{B}} + \left( {{\text{B}} - 1} \right) + 1} \right] \times {\text{M}} = \left( {2 \times {\text{B}}} \right) \times {\text{M}}$$

### Training and preparation of CNN model

Before the CNN model is officially used, it is necessary to train a large amount of data in the early stage to maximize the use of the model. 10 match videos of UEFA Women’s Champions League in 2021–2022 season are randomly selected, each video only captures 10 min of continuous match videos, and no fixed time level is set for the interception period. 10 intercepted videos are put into CNN model for training, and Table 2 shows the classification results of this training.

**Table 2:** Classification results of CNN model image features.

Image category	Total number of images	Correctly classified quantity	Number of misclassifications	Image classification accuracy (%)
Straight ball	1175	1170	5	99.57
Shoot in front of the door	585	582	3	99.49
Centers	1190	1188	2	99.83
Dribble	570	558	12	97.89
Set pieces	680	678	2	99.71
Break through	1395	1349	46	96.70
Direct angle (45 degrees)	465	462	3	99.35
Close shot (0 degrees)	575	552	23	96.00
Long shot angle (30 degrees)	870	848	22	97.47
Curved shot (15 degrees)	530	528	2	99.62
Header	1105	1104	1	99.91
Slide tackle	780	758	22	97.18
Volley shot	640	619	21	96.72
Long-range shot (40 degrees)	565	557	8	98.59
Low shot goal	875	867	8	99.09
Half-high shot goal	445	434	11	97.53
Cross shot goal	790	778	12	98.48
Penalty goal	520	518	2	99.62
Ultra long-range shot (50 degrees)	710	699	11	98.45
Quick counterattack goal	995	979	16	98.41

The above is a summary of the overall classification accuracy of 10 games, reflecting that CNN model has significant advantages in image feature classification. The model classifies a variety of different image categories, and the classification accuracy of almost every category exceeds 95%, and some even approach 99%. This shows the excellent ability of the model in distinguishing and classifying various football actions and goal angles. The model’s high accuracy and low number of wrong classifications show that it is very reliable in identifying different movements and goal angles. Table 3 sorts out and calculates the model training time, model training accuracy and model training loss value of 10 games respectively, as follows:

**Table 3:** Training results of CNN model single game.

Competition number	Model training time	Accuracy	Loss value
1	3 h 15 min	0.92	0.12
2	2 h 50 min	0.88	0.18
3	2 h 30 min	0.91	0.14
4	3 h 05 min	0.89	0.16
5	2 h 40 min	0.9	0.15
6	3 h 20 min	0.93	0.11
7	2 h 55 min	0.87	0.19
8	3 h 10 min	0.91	0.13
9	2 h 45 min	0.88	0.17
10	3 h 30 min	0.94	0.1

It can be clearly seen from the data in Table 2 that the CNN model has shown quite high accuracy in different games, and the accuracy of almost every game is above 88%. Meanwhile, the loss value is also maintaining at a relatively low level, indicating the stability and performance of the model. These results highlight the excellent performance of CNN model in classifying and training multiple games, and provide a reliable tool for the team to evaluate and improve the performance of players more accurately, which is expected to improve the winning rate of the game.

### System design of tactical analysis and evaluation of women’s football team based on CNN

Usually, football matches are 22-a-side matches, with 11 players on each side. The positions on the field can be divided into four layers, namely, striker, midfielder, defender and goalkeeper. The striker is the main goal scorer of the team. He is responsible for the attack and needs to have a breakthrough, speed up and dribble. Midfield is the main organizer of the team’s passing, and it is responsible for connecting the frontcourt and the defense. Midfield passing needs innovation and breakthrough, short passing should be decisive, and long passing should be accurate. The defender and the goalkeeper are the last line of defense of the team. The defender needs a certain ability to compete and his height needs to reach a certain standard. This is because the defender often uses the heading method. Goalkeepers seem to run less, but they need players’ high concentration throughout the game to prevent opponents from seizing the opportunity to score goals. Generally speaking, the time of a football match is 45 min in the first half and 45 min in the second half, with a 15 min intermission. The injury time is not necessarily determined according to the specific situation on the field. If it is in the form of final or late promotion, it will be added for 30 min on the basis of the original 90 min. If there is no winner during the extra time, it will enter the penalty time.

**Figure 3:**
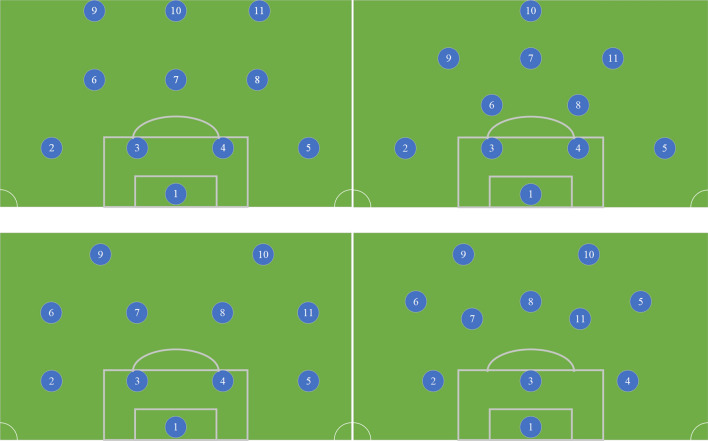
Bitmap of formation stations in common football matches.

In the same position, the women’s football team lacks certain physical confrontation ability compared with the men’s football team, so it needs certain skills in competition and defense, which requires players to have certain observation and analysis ability and control and coordination ability. Shooting requires the cooperation of frontcourt and midfield, which requires players to have certain passing innovation ability and understanding and judgment ability. On the field, the coach will make substitutions according to the situation on the spot and the players’ state. Substitution is not a simple position replacement. Sometimes it seems to be a simple substitution, but it is actually a change in formation and also a change in attack and defense. Common football formations are 4-2-3-1, 4-3-3, 4-4-2.3-5-2, etc.^26,27^. The specific station is shown in Fig. 3:

The overall time line of the football match is very long, which requires the players to have the physical quality to fight for a long time. Meanwhile, if the game lasts for 120 min without injury stoppage time, the players will have to withstand tremendous mental pressure and make a final confrontation with the goalkeeper one-on-one. It indicates that reasonable tactical arrangements are very necessary. In this paper, 11 starting players and 11 substitute players of a women’s team were selected to analyze their four abilities: observation and analysis, understanding and judgment, control and coordination, and passing innovation. First, the professional football coaching team is compared frame by frame, and the group that classified the images based on different ability levels is listed as the experimental group. Then, the data that are simply identified and classified without using CNN are used as the pre-segment group. Secondly, the data after classifying and outputting the video images of players’ usual training and recent matches by using CNN is used as the post-segment group, and after adding CNN, four abilities will be used as the classification criteria for classification and identification, and then output. Finally, the mean, standard deviation, standard error, minimum value, maximum value and the upper and lower limits of the 95% confidence interval of the mean of the four abilities are calculated according to the scoring system of the team. All experimental protocols have been approved by the Ethics Committee of Hangang University, and all methods have been carried out in accordance with relevant guidelines and regulations, with the informed consent of all subjects and/or their legal guardians.

This paper selects a women’s club in Hangzhou as the research object, and collects relevant data through the club’s official website and professional football platform. The researchers are all players who participated in training in the team or played for the team in 2021–2022. Everyone in the main team and the substitute team participates in the analysis of players’ personal recessive ability. For the analysis of players’ personal position mistakes on the field, the competition schedule selected in this paper is a match with a women’s football club in Dalian in September 2022, with a total duration of 102 min, 45 min in the first half and 7 min in injury time. In the second half, the game lasts 45 min and the injury time is 5 min. The result is 3–2. In the above two kinds of analysis, the scoring data related to the analysis of personal recessive ability comes from the unified scoring standard of the team, and the unified standard of the main team and the substitute team will eliminate most of the errors in the analysis process. The analysis of personal position mistakes comes from objective facts and is an electronic record of the actual content of the game. The running time, the number of mistakes and other related records have no influence on each other between the two groups. The specific personal data of all the subjects involved in this paper are shown in Table 4:

**Table 4:** Basic information of women’s football team players.

Main team			Substitute team		
Football clothing number	Position	Age	Football clothing number	Position	Age
Number 9	Striker	24	Number 30	Striker	22
Number 7	Striker	23	Number 29	Striker	24
Number 10	Striker	23	Number 13	Striker	21
Number 11	Midfielder	23	Number 28	Midfielder	22
Number 12	Midfielder	19	Number 24	Midfielder	16
Number 25	Midfielder	17	Number 22	Midfielder	20
Number 15	Defender	26	Number 14	Defender	22
Number 8	Defender	26	Number 2	Defender	21
Number 6	Defender	23	Number 5	Defender	20
Number 21	Defender	18	Number 4	Defender	19
Gatekeeper 1	Gatekeeper	29	Gatekeeper 18	Gatekeeper	25

It is necessary to define clear indicators and standards to evaluate the four personal hidden abilities of players: observation and analysis ability, understanding and judgment ability, control and coordination ability and passing innovation ability. Table 5 is a detailed description of each competency assessment method.

**Table 5:** Evaluation indicators of players’ personal recessive ability.

Personal recessive ability	Evaluation index	Index description
Observation and analysis ability (0–25 scores)	Observe the range	How wide a field can a player see during the running of the game?
	Observe the details	Whether the players can observe the relatively small details of the game
	Tactical analysis	Whether the players can make an accurate analysis of the tactical situation in the game
	Visual reaction time	The speed at which players react when they see a change in the game
	Spatial perception ability	Whether players can effectively perceive and use the space on the playing field
Understanding and judgment ability (0–25 scores)	Court situation judgment	Whether players can quickly judge the game situation and make correct decisions
	Tactical judgment	Whether the tactical choice of players in the game is wise
	Reaction rate	The reaction speed of players in the face of unexpected situations
	Risk judgment	Whether players can correctly predict risks and rewards when making decisions
	Interpretation of opponent’s action	Whether the player can effectively interpret the opponent’s actions and intentions
Control and coordination ability (0–25 scores)	Control of the ball	The player’s ability to master the ball, such as stopping the ball, dribbling, etc
	Motor coordination	Whether the players’ body coordination and movements are smooth in the game
	Teamwork	The degree of cooperation between players and teammates
	Motion accuracy	The accuracy and precision of a player's actions
	Balanced capacity	Whether the player can keep balance when doing dribbling-demanding actions
Passing innovation ability (0–25 scores)	Passing accuracy	Accuracy and precision of the player's passing
	Innovative passing	Whether the player can make innovative passes, such as long passes and cross passes
	Passing decision	Whether the player chooses the best passing target and is intercepted when passing the ball
	Passing power	The strength and speed of the player's passing, and the adaptability to different passing types
	Passing vision	Whether the player can see the passing opportunity and predict the running position of his teammates

## CNN model in tactical analysis and evaluation of women’s football team

### Analysis of CNN model on personal recessive ability of women’s football team players

**Figure 4:**
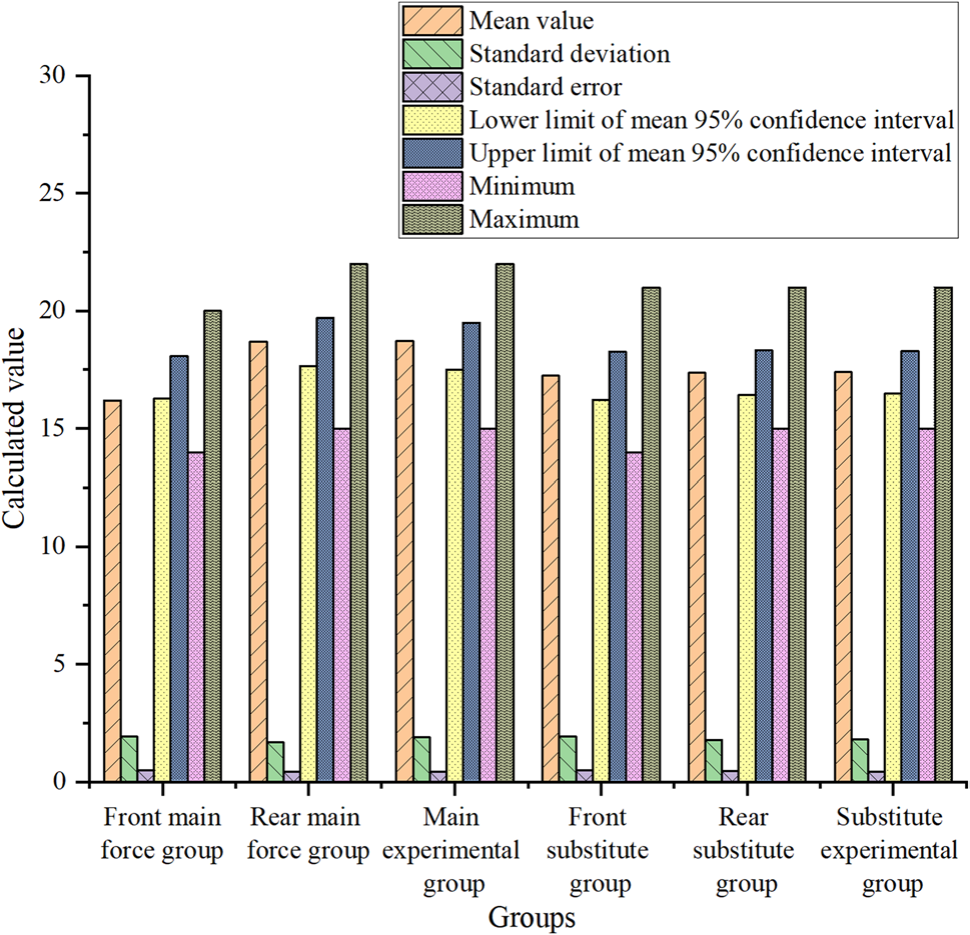
Schematic diagram of observation and analysis ability data.

The model designed according to the characteristics of CNN can help the team to analyze the players’ personal recessive ability more comprehensively. Seven values of the mean, standard deviation, standard error, minimum value, maximum value and upper and lower limits of the 95% confidence interval of the mean of the four abilities are calculated by a unified scoring standard. Through comparative observation, it clearly shows that the data of the post-paragraph group and the experimental group are very close. The analysis of players’ personal recessive ability includes four kinds, namely, observation and analysis ability, understanding and judgment ability, control and coordination ability and passing innovation ability. Among them, the specific data comparison of observation and analysis ability is shown in Fig. 4:

In Fig. 4, after classifying the pictures according to their ability types, three groups of related contents of observation and analysis ability are selected and scored, and then seven numerical values are calculated. It is found that for the main starting players, the standard deviation and standard error values of the pre-paragraph group, the post-paragraph group and the experimental group are basically the same, with little difference. The mean value, the upper and lower limits of the 95% confidence interval of the mean value, and the minimum and maximum values of the post-segment group and the experimental group are significantly higher than those of the pre-segment group, and the numerical differences between the post-segment group and the experimental group about these four groups are very small. For the substitute players, there is no obvious difference in other values except the minimum value among the pre-paragraph group, post-paragraph group and experimental group. This shows that it is closer to professional evaluation and more suitable for the main players to use the team’s scoring system to judge the players’ observation and analysis ability after extracting and classifying the video image features of the players’ usual training and recent games through CNN.

**Figure 5:**
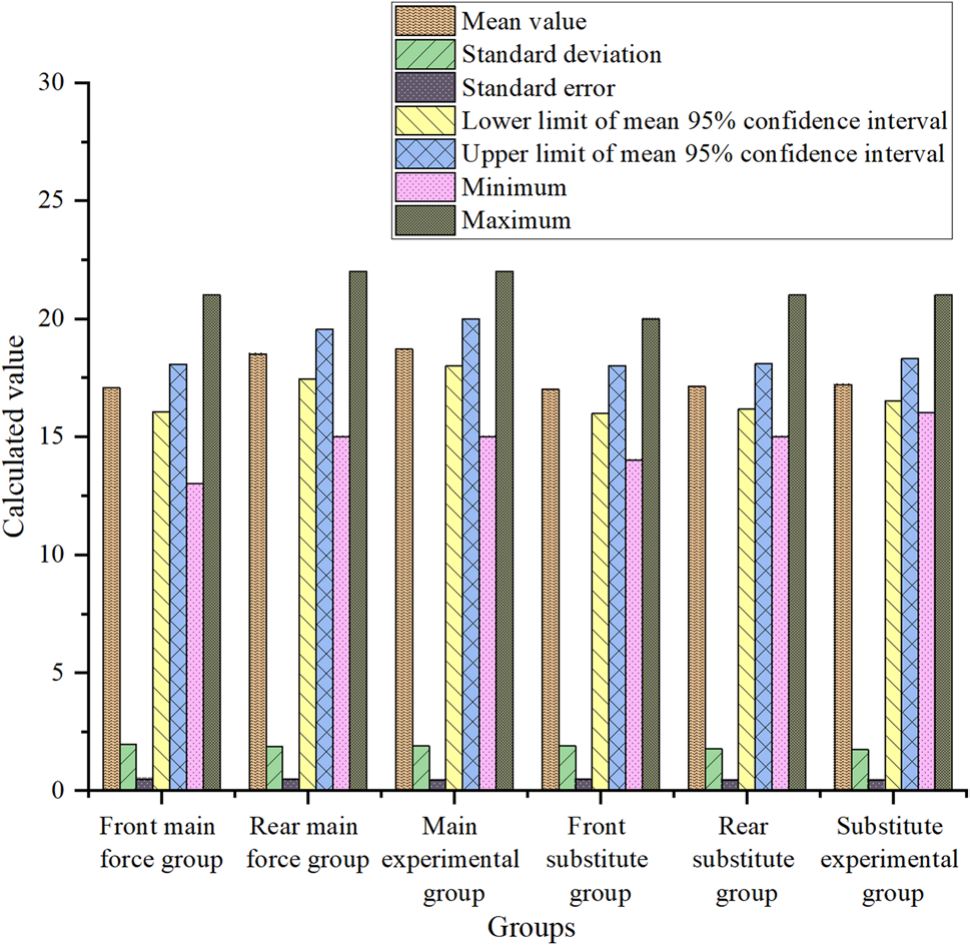
Schematic diagram of understanding and judging ability data.

Figure 5 shows the specific data of seven values of the mean, standard deviation, standard error, minimum value, maximum value and upper and lower limits of the 95% confidence interval of the mean value:

In Fig. 5, after classifying the pictures according to their ability types, three groups of related contents of comprehension and judgment ability are selected and scored, and then seven numerical values are calculated. It is found that for the main starting players, the standard deviation and standard error values of the pre-paragraph group, the post-paragraph group and the experimental group are basically the same, with little difference. The mean value, the upper and lower limits of the 95% confidence interval of the mean value, and the maximum and minimum values of the post-segment group and the experimental group are significantly higher than those of the pre-segment group, and the numerical differences between the post-segment group and the experimental group about these four groups are very small. For the substitute players, there are slight differences in the upper and lower limits of the 95% confidence interval of the average value among the pre-paragraph group, the post-paragraph group and the experimental group, and there are obvious differences between the maximum value and the minimum value, but there are no obvious differences in other values. This shows that it is closer to professional evaluation and more suitable for the main players to use the scoring system of the team to analyze and judge the players’ understanding and judgment ability after extracting and classifying the video image features of the players’ usual training and recent games through CNN.

**Figure 6:**
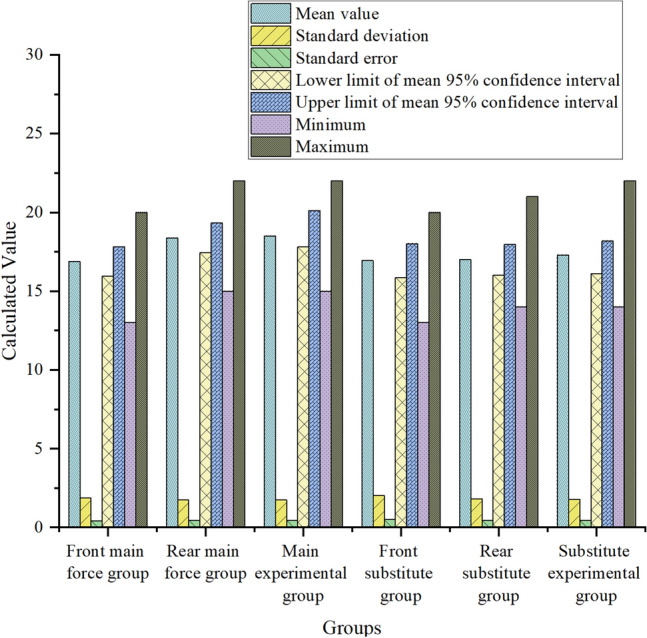
Schematic diagram of control coordination ability data.

The specific data of seven numerical values, such as mean value, standard deviation, standard error, minimum value, maximum value and upper and lower limits of 95% confidence interval of mean value, of control coordination ability are shown in Fig. 6:

In Fig. 6, after classifying the pictures in terms of ability types, three groups of related contents of control and coordination ability are selected and scored, and then seven numerical values are calculated. It is found that for the starting main players, the differences in judging and calculating the maximum and minimum values of the three groups of pre-paragraph group, post-paragraph group and experimental group are the most obvious, and the differences between the data of post-paragraph group and experimental group are kept at about 2. Comparing the first two numerical values of ability, it is found that the differences in maximum values are the most obvious. For the substitute players, there is no significant difference among the pre-paragraph group, post-paragraph group and experimental group except for the maximum value. This shows that the CNN is used to extract and classify the video image features of the players’ usual training and recent matches, and then the team’s scoring system is used to analyze and judge the players’ control and coordination ability. No matter the starting main players or the substitute players, the change of the maximum value is the most obvious.

**Figure 7:**
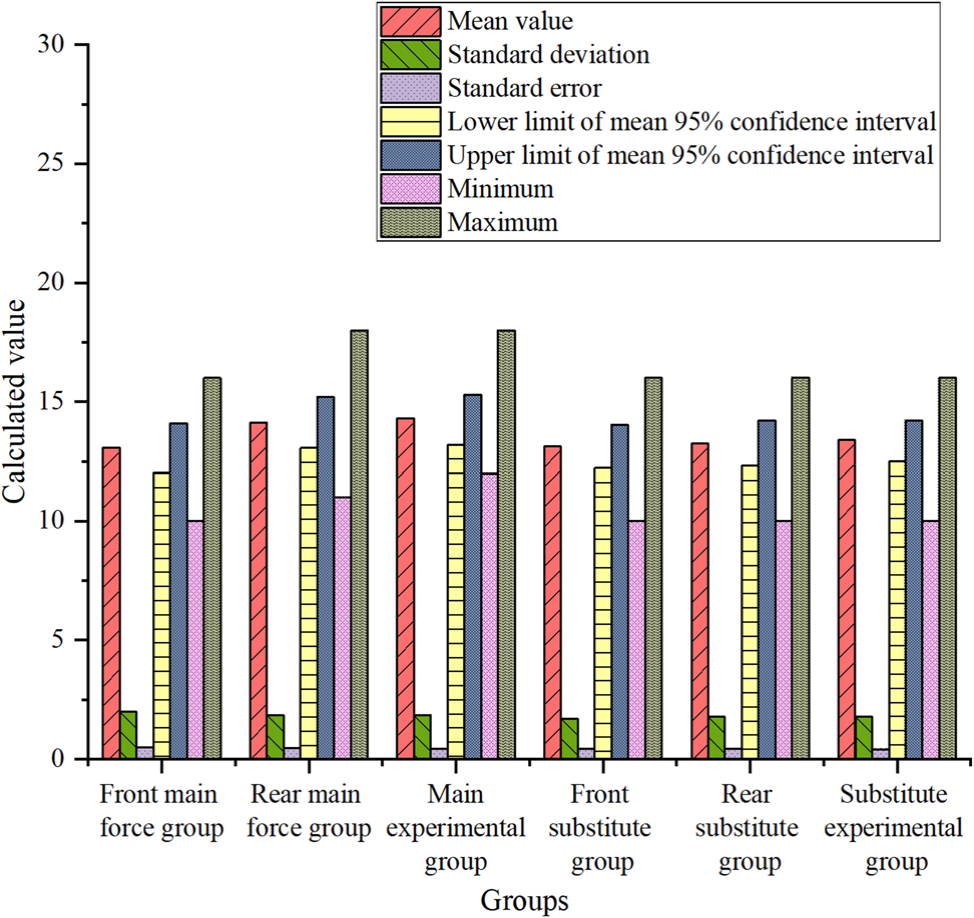
Schematic diagram of passing innovation ability data.

The specific data of seven numerical values, i.e., mean, standard deviation, standard error, minimum value, maximum value and upper and lower limits of 95% confidence interval of mean value, of passing innovation ability are shown in Fig. 7:

In Fig. 7, after classifying the pictures according to their ability types, seven numerical values are calculated after selecting three groups of related contents of passing innovation ability. It is found that for the main starting players, there are slight differences in standard errors among the three groups: the pre-paragraph group, the post-paragraph group and the experimental group, and the upper and lower limits of the mean value and the 95% confidence interval of the mean value are small, but the maximum value is obvious. For the substitute players, the upper and lower limits of the 95% confidence interval of the mean, standard deviation, standard error, minimum value, maximum value and mean value in the post-paragraph group and the experimental group are basically the same, and they are slightly higher than those in the pre-paragraph group with slight differences. This shows that there is no obvious difference in the overall changes, but there are slight numerical changes after the CNN extracts and classifies the image features of players’ usual training and recent games, and then uses the team’s scoring system to analyze and judge the players’ passing innovation ability.

### CNN model analysis of personal position errors of women’s football team players on the field

CNN can not only judge the individual ability more accurately according to the player’s usual personal performance before the game, but also play a great role in the game. CNN can be used to monitor the players’ state in real time, and provide a summary of players’ personal mistakes through real-time identification, so that coaches can better cope with the changes on the field. In this paper, by extracting the field data of a women’s team league match, the error statistics of players in different positions are completed by using CNN.

**Figure 8:**
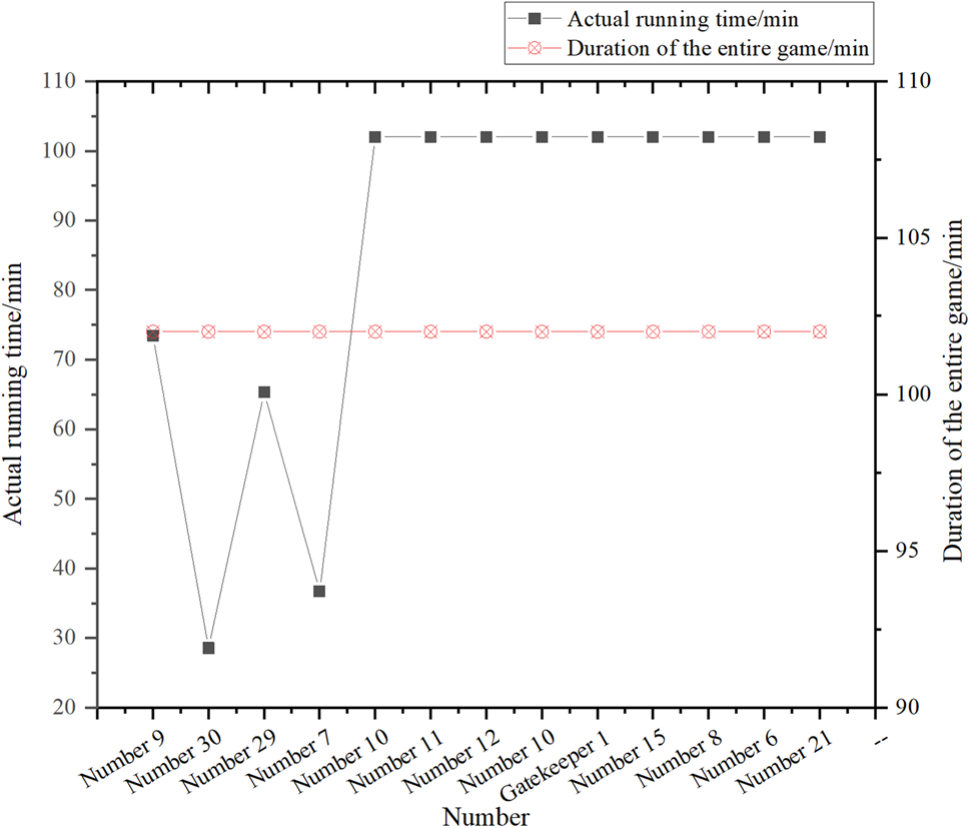
Time chart of all players’ individual full-court running.

**Figure 9:**
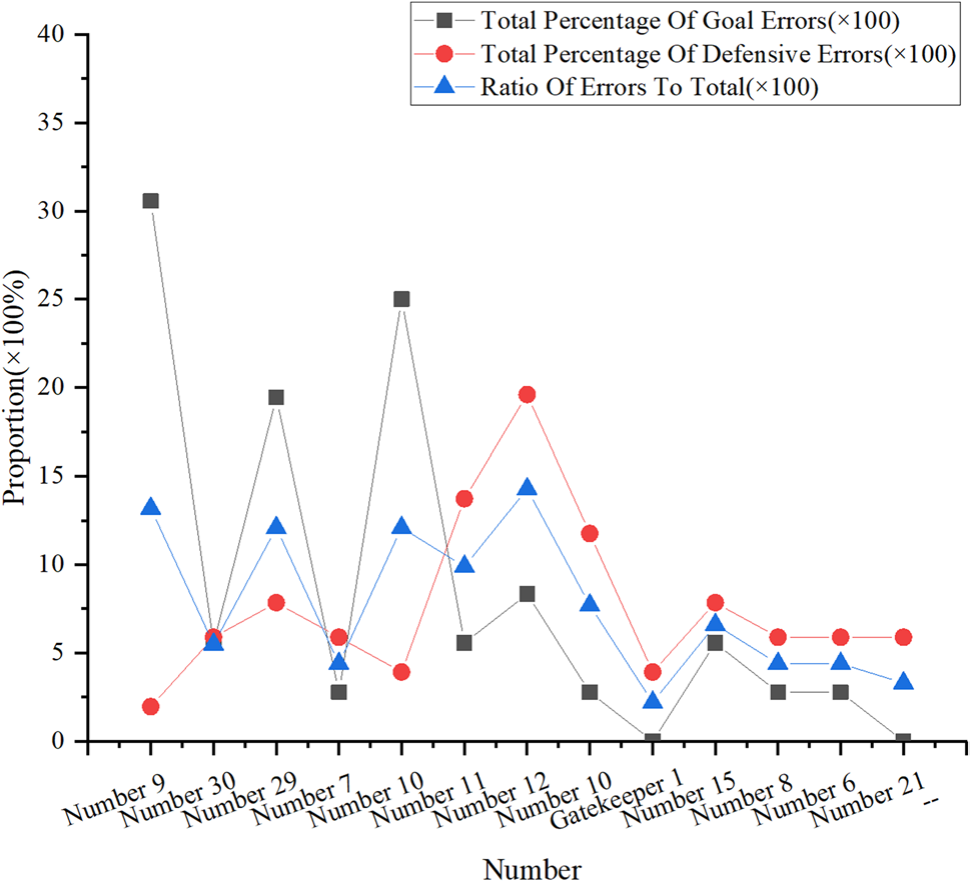
Proportion of individual game mistakes of all players on the court.

According to statistics, No. 9 is the left striker, No. 30 is the backup left striker, No. 29 is the right striker, No. 7 is the backup right striker, No. 10 is the center, No. 11 is the front waist, No. 12 is the central defender, No. 25 is the back waist, No. 1 is the goalkeeper, and the four defensive players are No. 15, No. 8, No. 6 and No. 21 respectively. Among them, the individual running time and actual competition time of all players are shown in Fig. 8:

By analyzing the scoring mistakes and ball-keeping mistakes of all players in the whole game, it is concluded that the proportion of individual mistakes of players is as shown in Fig. 9:

In Figs. 8, 9, with the passage of time, there are more and more goal mistakes and ball-keeping mistakes of left and right forwards, especially goal mistakes, which greatly affect the team’s goal efficiency. Football matches need to run all the time, especially in the forward position, and need to speed up and break through the opponent’s defense line to score goals. By observing the numerical value in Fig. 9, it is found that the individual goal mistakes account for about 20%, the individual ball-keeping mistakes account for nearly 10% and the individual total mistakes account for about 12% in the running time of the right striker. The left striker’s personal goal mistakes account for about 30% of the total, personal ball-keeping mistakes account for less, and personal total mistakes account for about 13%. In addition, among the players who have a high proportion of other mistakes, the number 25 player in the lower back has a high proportion of ball-keeping mistakes, and the personal ball-keeping mistakes account for nearly 12% of the total; The number 10 player’s personal goal mistakes account for 25% of the total. After running for about 65 min on the 29 th right striker, the coach changed to the 7th right striker; After the left striker No.9 ran for about 73 min, the coach made a substitution measure and replaced him with the substitute left striker No.30. The coach at the center position and the back position did not make any substitutions. This shows that the statistics of players’ personal mistakes based on CNN can help coaches to make coping strategies more quickly, and coaches can clearly see the personal level of members from the statistics, and combine the analysis of personal hidden ability before the game to arrange the most suitable players to play in the right time according to the players’ personal ability to stabilize the situation.

## Discussion

In the latest research on individual ability of women’s football, İslam selected 307 players from Turkish women’s football league in 2021–2022 season, and adopted Suo Beier test and intermediary analysis. The research results support four hypotheses: (1) Sports psychological ability has a positive impact on psychological energy; (2) Psychological skill level affects athletes’ courage level; (3) Sports psychological energy has a positive effect on courage; (4) Sports psychological energy plays an intermediary role between psychological skills and courage. This emphasizes the importance of cultivating female football players’ psychological skills and energy^28^. Another study, conducted by López-Valenciano et al., reviewed 22 studies on the injury rate of women’s football. The results showed that the total injury rate of female football players was 6.1 times per 1000 h, and the incidence of competition injuries was six times that of training injuries, which were 19.2 times and 3.5 times per 1000 h respectively. Lower extremity injuries were the most common, and muscle/tendon and joint/ligament injuries were the most common, which were usually related to traumatic events^29^. This highlights the risk of injury faced by female football players, and measures need to be taken to reduce this risk. The unity of this research and the above research is reflected in the fact that all three parties pay attention to the health and safety of female football players, emphasizing the importance of female players’ psychological quality, skill level and injury risk. The innovation of this paper lies in the combination of CNN technology and the special needs of women’s football, which not only helps to improve the efficiency of personal skill evaluation, but also reduces the analysis time, thus better meeting the needs of women’s football training and competition. This quantitative evaluation combined with multiple ability indexes can provide a novel method for professional training and performance improvement in the field of women’s football, which has important practical application potential.

Although the research in this paper has made some valuable achievements, there are still some shortcomings. The shortcomings of this paper are that the classification of players’ personal hidden abilities is only divided into four types: observation and analysis ability, understanding and judgment ability, control and coordination ability and passing innovation ability, and the classification of abilities before the actual game can be more detailed. In addition, when analyzing the players’ mistakes in different positions on the field, only the players’ mistakes are considered, and other possible situations on the field are not considered, such as injuries, penalties, and even the physiological effects of female players at special times. In this case, it is bound to be imperfect, and more detailed division research is needed in the future to continuously improve the research results. The purpose of this paper is to help the women’s football team complete the pre-match tactical training, reduce the analysis time of players’ mistakes in the game, deal with different opponents in the game and improve the winning rate.

### Ethical approval

All experimental protocols have been approved by the Ethics Committee of Hangang University, and all methods have been carried out in accordance with relevant guidelines and regulations, with the informed consent of all subjects and/or their legal guardians.

## Conclusion

In order to extract and classify the players’ characteristics from the images of multi-frequency frame changes and complete the tactical plan more conducive to the game, this paper puts forward a method of tactical analysis and evaluation of women’s football team based on CNN, which analyzes the players’ hidden ability before the game and the players’ mistakes in different positions on the field. For the analysis of personal recessive ability before the game, firstly, after comparing the professional football coaches frame by frame, the group that classifies the pictures according to their different abilities is listed as the experimental group. The data that are not simply identified and classified by CNN are used as the pre-segment group; Using CNN to classify and output the video images of players’ usual training and recent competition as the post-segment group. Secondly, using the team’s scoring system to calculate the mean, standard deviation, standard error, minimum value, maximum value and the upper and lower limits of the 95% confidence interval of the mean value of the four abilities. Finally, the size and variation differences of seven values of three groups of data are compared. Before the formal test, this paper trained 10 randomly selected 10 min matches from the UEFA Women’s Champions League in 2021–2022. The results show that the model has excellent accuracy in the classification of image features of various football movements and goal angles, and the classification accuracy of almost every category is as high as 95%. In addition, the accuracy of the model is above 88% for each individual match, which highlights the reliability and consistent stability of its identification and classification in women’s football matches.

The test results show that the scores of players’ observation and analysis ability, understanding and judgment ability, control and coordination ability and passing innovation ability are all close to those of the experimental group. The error of average value is less than 0.5, the error of standard deviation is less than 0.1, the error of standard error is less than 0.1, the error of minimum value and maximum value is less than 1, and the upper and lower limits of 95% confidence interval of average value are less than 0.5. The smaller the error, the closer it is to professional evaluation. According to the analysis of the players’ mistakes in different positions on the field, the two players in the forward position made the highest mistakes, which affected the team to score and keep the ball and were replaced by substitute players at 73.44 min and 65.28 min respectively. After the substitute players played, the team’s forward position mistake rate was significantly reduced. The above conclusion shows that it is closer to professional evaluation to extract and classify the video images of players’ usual training and recent games through CNN, and then evaluate the players’ personal recessive ability in combination with the team’s scoring system. The application of CNN helps to provide more accurate evaluation results. The statistics of players’ personal mistakes based on CNN not only provides a real-time analysis tool for the coach team, but also helps the coach to formulate coping strategies more quickly. This result highlights the important role of the CNN model formed by testing in accuracy, and provides a reliable performance evaluation and real-time analysis tool for the team.

### Supplementary Information

Supplementary Tables.

## Data Availability

All data generated or analysed during this study are included in this published article [and its supplementary information files]. If someone wants to request the data from this study please contact the Corresponding author (Guoqin Jiang, 931232166@stu.jhun.edu.cn).
